# Withholding the Annual Report: An Explanation Asked for

**Published:** 1916-05-27

**Authors:** 


*To the Editor of* THE HOSPITAL.

### Withholding the Annual Report: An Explanation Asked For.

*To the Editor of* THE HOSPITAL.

SIR,—With reference to the letter from Charing Cross Hospital in your issue this week concerning the printing, etc., of annual report, and to your remarks in the "News," it would be interesting to learn how between £200 and £300 can be saved by not sending the report to the subscribers except by request.

The principal expense is in the "setting up" and printing, and not in the number of copies supplied. Incidentally I notice that the total of official printing, etc., and postages for Charing Cross Hospital in 1914 (I have not the 1915 figures) is only £118, and this must include other items in addition to annual report. Is one to assume that the enormous saving is on "Appeals"?—I have the honour to be, your obedient servant,

May 20, 1916. ASSISTANT SECRETARY.

